# Aberrant structural and functional alterations in postpartum depression: a combined voxel-based morphometry and resting-state functional connectivity study

**DOI:** 10.3389/fnins.2023.1138561

**Published:** 2023-05-25

**Authors:** Chunlian Chen, Bo Li, Shufen Zhang, Zhe Liu, Yu Wang, Minghe Xu, Yuqing Ji, Shuang Wang, Gang Sun, Kai Liu

**Affiliations:** ^1^Jinzhou Medical University, Jinzhou, China; ^2^Department of Radiology, The 960th Hospital of the PLA Joint Logistic Support Force, Jinan, Shandong, China; ^3^Department of Obstetrics, Shandong Second Provincial General Hospital, Jinan, China

**Keywords:** postpartum depression, functional connectivity, voxel-based morphometry, structural MRI, Resting-State fMRI

## Abstract

**Objectives:**

Postpartum depression (PPD) is a severe postpartum psychiatric disorder with unclear pathogenesis. Previous neuroimaging studies have reported structural or functional alterations in areas associated with emotion regulation, cognitive disorder, and parenting behaviors of PPD. The primary goal of this investigation was to explore the presence of brain structural alterations and relevant functional changes in PPD patients.

**Methods:**

A total of 28 patients and 30 matched healthy postnatal women (HPW) underwent both three-dimensional T1-weighted magnetic resonance imaging (MRI) and resting-state functional MRI. Structural analysis was performed by voxel-based morphometry (VBM), followed by resting-state functional analysis using a seed-based whole-brain functional connectivity (FC) approach with abnormal gray matter volume (GMV) regions as seed.

**Results:**

Compared with HPW, the PPD patients showed increased GMV in the left dorsolateral prefrontal cortex (DLPFC.L), the right precentral gyrus (PrCG.R), and the orbitofrontal cortex (OFC). In the PPD group, the DLPFC.L showed increased FC with the right anterior cingulate and paracingulate gyri (ACG.R) and the right middle frontal gyrus (MFG.R); the FC between the PrCG.R and the right median cingulate and paracingulate gyri (DCG.R) exhibited enhanced; the OFC showed increased FC with MFG.R and the left inferior occipital gyrus (IOG.L). In PPD, GMV of DLPFC.L was positively correlated with EDPS scores (*r* = 0.409 *p* = 0.031), and FC of PrCG.R-DCG.R was positively correlated with EDPS scores (*r* = 0.483 *p* = 0.020).

**Conclusion:**

Structural and functional damage of the DLPFC.L and OFC is associated with cognitive disorders and parenting behaviors in PPD, while structural abnormalities of the DLPFC.L and PrCG.R are involved in impaired executive function. The increased GMV of DLPFC.L may be a unique structural pathological mechanism of PPD related to the inability of PPD patients to withstand long-term parenting stress. These findings have important implications for understanding neural mechanisms in PPD.

## 1. Introduction

Pregnancy and childbirth is often a positive and joyous event for most women and their families. However, for some women, major changes in physiology, psychology, emotion, and social role in the postpartum period can lead to negative outcomes such as mental disorders. Postpartum depression (PPD) is the most common psychiatric disorder during the postpartum period (Tainaka et al., 2022). PPD is considered as a common social and mental health problem, and women with PPD can suffer from some symptoms, such as depressed mood, loss of interest, anxiety, sleep disorders, insomnia, irritability, and compulsive behavior, usually accompanied by corresponding thought-language and psychomotor disorders (Stewart and Vigod, 2019). Typically, PPD occurs within 4–6 weeks after childbirth, but it may last several months or even a year (Alshikh Ahmad et al., 2021; Estiningtyas et al., 2021). Recently, it was found that PPD may affect up to 30% of all women after delivery (Alshikh Ahmad et al., 2021), and the estimated prevalence of it ranged from 14.3% to 19.3% in China (Qiu et al., 2020). PPD can negatively affect maternal physical and mental health, which also can lead to difficult breastfeeding and mother-infant connection, resulting in a lack of attachment between the mother and the baby, and some women with PPD even have thoughts of self-harm and infanticide (Laufer, 2021). It has been shown that PPD can indirectly affect the emotional and intellectual development of infants and even increase the risk of depression in their period of adolescence (Aoyagi and Tsuchiya, 2019). PPD has been identified as one of the most severe global public health issues because of its high prevalence and detrimental consequences in the last decade (Wan Mohamed Radzi et al., 2021; Chen Q. et al., 2022). However, the physiological, pathological, and psychosocial mechanisms which contribute to the development of PPD remain poorly understood.

Neuroimaging techniques are considered excellent tools playing an essential role in identifying cerebral neuropathology abnormalities in multiple diseases (Chen Z. et al., 2022; Picó-Pérez et al., 2022) and have been recently applied to investigate the structural and functional abnormalities of the brain in PPD. Relatively few studies have been conducted on structural changes in the brain of PPD patients, mainly focusing on white and gray matter. A diffusion tensor imaging (DTI) study found that PPD showed significantly increased fractional anisotropy (FA) and axial diffusivity (AD) in the right anterior thalamic radiation (ATR) tract, significantly increased FA, and reduced radial diffusivity (RD) in the cingulum tract in patients compared with HPW (Long et al., 2023). A surface-based morphometry (SBM) study found that PPD patients showed a thinner cortical thickness in the right inferior parietal lobule compared with the healthy controls and increased surface area was observed in the left superior frontal gyrus, caudal middle frontal gyrus, middle temporal gyrus, insula, and right supramarginal cortex in PPD patients (Li et al., 2021), and a voxel-based morphometry (VBM) study found that PPD patients had increased regional gray matter volume (GMV) in the left dorsolateral prefrontal cortex (DLPFC.L) and right anterior insula (anI) relative to HPW (Cheng B. et al., 2022). Additionally, some functional magnetic resonance imaging (fMRI) studies have revealed that PPD patients exhibit altered neural function in brain networks involved in default mode network (DMN), salience network (SN), executive control network (ECN), sensorimotor network (SMN), reward network, and limbic system (LIN), which involved the DLPFC, anterior/posterior cingulate cortex (ACC/PCC), orbitofrontal cortex (OFC), medial prefrontal cortex, amygdala, hippocampus, temporal cortices, insular, striatum, middle frontal gyrus (MFG) and may underlie the deficits in cognitive control, emotional regulation, affective processing, reward processing, and visuospatial and body-signal integration (Mao et al., 2020; Zhang et al., 2020; Cheng et al., 2022; Zhang S. et al., 2022). However, most of these studies of PPD have focused on either structural or functional changes rather than both. The VBM is a comprehensive brain structural analysis technique that can assess anatomical changes in the brain through quantitative calculations and analysis (Lv et al., 2023). Functional magnetic resonance imaging (fMRI) is a noninvasive neuroimaging technique that enables the identification of the brain regions and networks underpinning cognitive tasks, which can detect changes in functional connectivity (FC) between brain regions related to neurocognitive processes (Hay et al., 2022). The seed-based analysis is a model-based approach that relies on defining a particular ROI or set of ROIs and correlating the BOLD fMRI time series of this region against the time series of all other regions, resulting in a functional connectivity map to assess neural network reconfiguration (Bonzano et al., 2022). The combined VBM and FC techniques, which have been used in brain studies for diseases such as obsessive compulsive disorder (OCD) (Xu et al., 2023), vestibular migraine (VM) (Zhe et al., 2021), and bulimia nervosa (BN) (Li et al., 2022) may be a potential method to explore the neurobiological mechanisms of PPD thought to investigate the effects of regional GMV changes on whole-brain functional integrity. Nevertheless, no studies have investigated coexisting structural and functional differences in patients with PPD.

We conducted three hypotheses for the present study: we tried to apply the VBM technique to find out whether there were GMV changes in PPD patients. If so, we took these altered brain structural areas as seed regions for whole-brain FC analysis. Moreover, the correlation analysis was performed to explore the relationship between significant cerebral regions and clinical characteristics.

## 2. Materials and methods

### 2.1. Participants

A total of 28 PPD patients and 30 HPW were recruited from the Department of Obstetrics of the 960th Hospital of the PLA Joint Logistics Support Force and the Department of Obstetrics of Shandong Second Provincial General Hospital. HPW matched with the PPD group in terms of age and education level. In total, two experienced senior psychiatrists determined the diagnosis of PPD using the Structured Clinical Interview for Diagnostic and Statistical Manual of Mental Disorders, fifth edition (DSM-V) and Chinese Classification and Diagnostic Criteria of Mental Disorders, third edition (CCMD-3). Further inclusion criteria for the PPD group were as follows: (a) ages ≥20 years, in the sixth week after delivery (healthy full-term infants), (b) the first onset without any treatment, (c) Edinburgh postpartum depression scale (EPDS) scores ≥12, and (d) right-handedness. The exclusion criteria were the following: (a) suffering from serious neurological or mental disorders other than PPD, (b) substance abuse or dependence, (c) first-degree relatives had psychiatric disorders, (d) prior miscarriage or pregnancy losses, (e) prematurity and the infant of low-birth weight, (f) history of head trauma and intracranial tumor, (g) poor imaging quality or head motion, and (h) organic abnormalities for MRI routine series. The inclusion criteria for the HPW group were as follows: (a) ages ≥20 years, in the 6th week after delivery (healthy full-term infants), (b) no current or previous history of depressive episode, (c) no sedative, anesthetic, or analgesic drugs were taken, (d) EPDS score <3, and (e) right-handedness. The exclusive criteria were the same as those in the PPD group.

All the enrolled participants accepted questionnaires in the 6th week after delivery that enabled the gathering of personal information and scores on the EPDS and Pittsburgh Sleep Quality Index (PSQI). The ethics committee of the 960th Hospital of the PLA Joint Logistics Support Force approved the present study, complying with the ethical standards of the Declaration of Helsinki. After a complete written and oral explanation of the experimental procedures and objectives and relevant contraindications of the study, written informed consent was obtained from all participants.

### 2.2. MR imaging acquisition

All MRI data were acquired on a 3.0 T MR system (Discovery MR750, General Electric, Milwaukee, WI, United States) equipped with a standard eight-channel phased-array head coil. Before scanning, patients wore earplugs to reduce the noise of the scanner. Sponge pads were fixed around all participants' heads during scanning to minimize motion while maintaining the supine position. Every participant performed the following behaviors: keep quiet, stay awake, and keep eyes closed without thinking.

High-resolution structural T1-weighted scan (three-dimensional brain volume, 3D BRAVO) were performed with the following parameters: time repetition (TR) = 8.2 ms; time echo (TE) = 3.2 ms; flip angle = 12; field of view (FOV) = 240 mm × 240 mm; slices = 115; voxel size = 1 mm; and thickness = 1.0 mm. Resting-state blood oxygenation level-dependent (BOLD) MR images were performed with the following parameters: TR = 2,000 ms; TE = 30 ms; flip angle = 90; FOV = 240 mm × 240 mm; matrix = 64 × 64; slice thickness = 4.0 mm, no interspace; the number of slices = 41; gradient echo-planar volumes = 200; and duration was 6 min 40 s.

### 2.3. Data processing and analysis

#### 2.3.1. VBM data

Structural data preprocessing and statistical analyses were performed using the Statistical Parametric Mapping 8 (SPM8, http://www.fil.ion.ucl.ac.uk/spm/) and its embedded voxel-based morphometry (VBM8, http://dbm.neuro.uni-jena.de/vbm8/) toolbox on the MATLAB (The Math-Works Inc., Natick, MA, USA). The following steps include: Firstly, the format of images was converted from DICOM to NIFTI and spatial registration of T1-weighted images to a reference brain template, and then the segmentation of images into the gray matter (GM), white matter (WM), and cerebrospinal fluid (CSF). Then, the resulting images were registered to standard Montreal Neurological Institute (MNI) space (http://www.mni.mcgill.ca/). Spatial registration was normalized to MNI space using the high-dimensional diffeomorphic anatomic registration through the exponentiated lie algebra (DARTEL) method with a voxel size of 1.5 × 1.5 × 1.5 mm^3^. Finally, smoothing was done using a Gaussian kernel with a 4 mm full-width at half maximum (FWHM) Gaussian kernel.

#### 2.3.2. FMRI data

Functional data preprocessing and statistical analyses were performed by using SPM8 and the Resting-State fMRI Data Analysis Toolkit (REST, http://www.restfmri.net) on MATLAB. The steps include those explained as follows: the first 10 time points of data were removed to eliminate the effects of inadaptability and magnetic field inhomogeneity. Then, the slice timing step was performed, and rigid body motion data correction steps were performed by artificially removing data in which head motion and rotation was >1.5 mm or >1.5. The remaining dataset was spatially normalized to the MNI template. After normalization, all images were resampled into 3 × 3 × 3 mm^3^ and spatially smoothed with a 4 mm FWHM Gaussian kernel. The effects of high-frequency signal, low-frequency drift, and physiological noise were removed by bandpass filtering (0.01–0.08 Hz) and linear drift.

#### 2.3.3. Seed-based-GMV of functional connectivity analysis

The significantly different GMV regions in patients with PPD (compared with HPW) were selected as a seed site. FC maps were obtained by calculating the correlation coefficient between the mean time series of each seed site within the whole brain. Then, the correlation coefficient maps were converted into Fisher-z maps by bivariate Fisher's z-transform to improve normality.

#### 2.3.4. Statistical analysis

Demographic variables and clinical data analyses were performed using the Statistical Package for Social Sciences (SPSS, 23.0) software. In total, two sample t-tests were used to compare age, education level, family income, EPDS score, and PSQI score between the PPD and HPW groups. The method and timing of delivery between the two groups were analyzed by the chi-square (χ^2^) test. Statistically significant correlation thresholds were set at a p-value of 0.05.

Voxel-based significant differences in GMV and FC strength between the patients with PPD and HPW were analyzed using SPM 8 software. Correction for multiple comparisons was performed using a Gaussian random field (GRF-corrected, voxel *p* < 0.05). The correlation analyses were carried out between a GMV value/FC _Seed−Based−GMV_ value and the EPDS/PSQI scores, exploring the association between structural/functional abnormalities and global cognitive function in PPD patients (*p* < 0.05 was statistically significant).

## 3. Results

### 3.1. Demographic and clinical characteristics

The demographic and clinical characteristics of all participants were displayed in Table 1. The status of age, delivery method and time, feeding options, education level, and family income showed no significant differences between the PPD and HPW groups (*p* > 0.05). The PPD group showed significantly higher EPDS and PSQI scores than HPW (*p* < 0.001).

**Table 1:** Demographic and clinical characteristics of participants.

	**PPD (*****n*** = **28)**		**HPW (*****n*** = **30)**		
**Characteristics**	**Mean (SD)**	**Percent (%)**	**Mean (SD)**	**Percent (%)**	***p-*** **value**
Age (year)	29.75 ± 4.49		28.46 ± 4.64		0.29^a^
Primipara	24	85.71	25	83.33	0.80^b^
Cesarean	3	10.71	6	20.00	0.49^b^
Breastfeeding	28	100	30	100	
Education (years)	13.96 ± 2.02		13.30 ± 2.11		0.22^a^
EPDS	16.64 ± 2.12		0.40 ± 0.72		0.00^a^
PSQI	14.50 ± 3.10		5.86 ± 2.45		0.00^a^
Family income	13.50 ± 3.34		13.17 ± 2.26		0.66^a^

SD, standard deviation; EPDS, Edinburgh postpartum depression scale;

a independent-sample t tests;

b χ^2^. Age, years; Education level, years; Family income, thousand RMB.

### 3.2. VBM results

**Figure 1:**
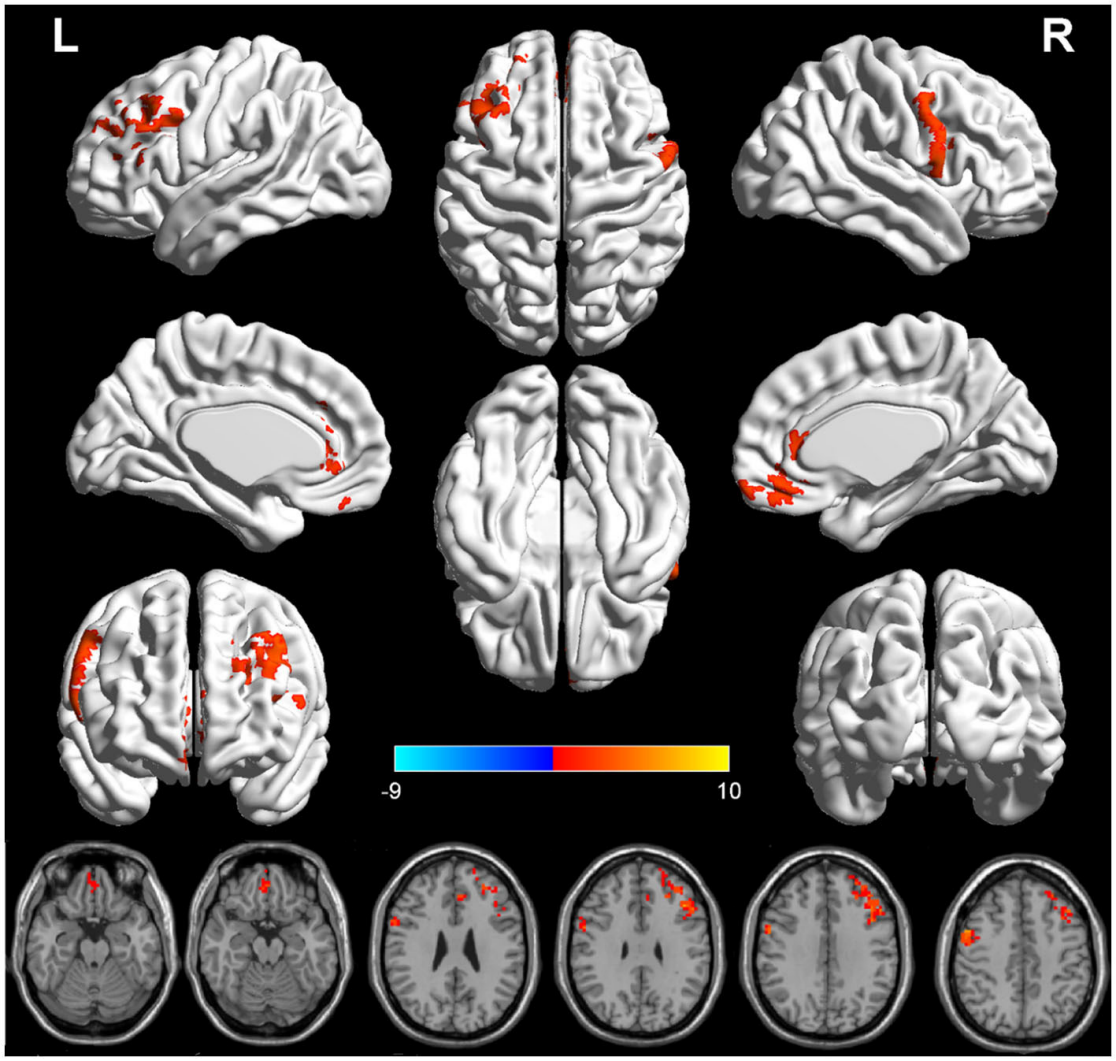
The red region indicated larger GMV in postpartum depression patients (PPD) than in the healthy postnatal women (HPW). GMV, gray matter volume; DLPFC.L, the left dorsolateral prefrontal cortex;PrCG.R, the right precentral gyrus; OFC, the orbitofrontal cortex.

There were significant differences in GMV between the two groups in three brain regions (Figure 1 and Table 2). Compared with HPW, the left dorsolateral prefrontal cortex (DLPFC.L), the right precentral gyrus (PrCG.R), and the orbitofrontal cortex (OFC) showed significantly increased volume in PPD patients.

**Table 2:** GMV differences between PPD patients and HPW.

		**MNI**				
**Cluster regions**	**Volume**	**X**	**Y**	**Z**	**Cluster size (Voxels)**	**Peak intensity**
DLPFC.L	Increase	−45	24	42	224	3.94
PrCG.R	Increase	51	6	45	162	3.86
OFC	Increase	3	63	−15	139	3.08

GMV, gray matter volume; L, left; R, right; MNI, Montreal Neurological Institute.

### 3.3. Seed-based functional connectivity results

**Figure 2:**
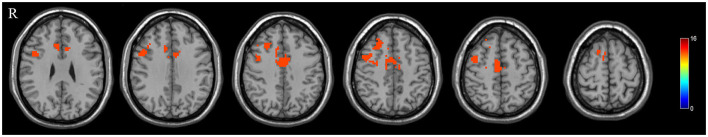
The red region indicated increased FC in postpartum depression patients (PPD) compared with the healthy postnatal women (HPW). ACG.R, the right anterior cingulate and paracingulate gyri; MFG.R, the right middle frontal gyrus; DCG.R: the right median cingulate and paracingulate gyri; IOG.L, the left inferior occipital gyrus. Figure C, functional connectivity. FB, FC between the DLPFC.L and ACG.R, MFG.R.

**Figure 3:**
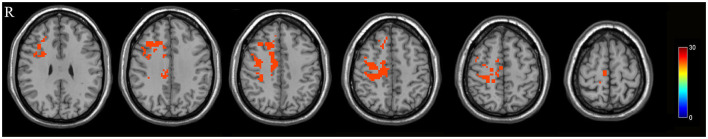
FC between the PrCG, R and DCG.R.

**Figure 4:**
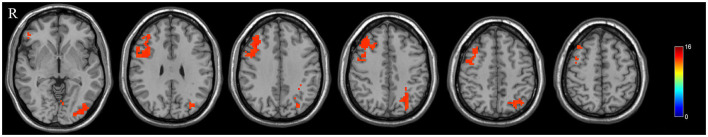
FC between the OFC and MFG.R, IOG.L.

The DLPFC.L, PrCG.R, and OFC were significant differences regions of GMV between the PPD patients and HPW, which were selected as seed regions for whole-brain functional connectivity analysis. In the PPD group, the DLPFC.L showed increased FC with the right anterior cingulate and paracingulate gyri (ACG.R) and the right middle frontal gyrus (MFG.R); the FC between the PrCG.R and the right median cingulate and paracingulate gyri (DCG.R) exhibited enhanced; the OFC showed increased FC with MFG.R and the left inferior occipital gyrus (IOG.L) (Figures 2–4 and Table 3).

**Table 3:** Significant differences in FC between PPD patients and HPW.

		**MNI**				
**Seed regions**	**Areas with altered FC**	**X**	**Y**	**Z**	**Cluster size (Voxels)**	**Peak intensity**
DLPFC.L	Cingulum_Ant_R	6	30	18	414	10.66
	Frontal_Mid_R	42	3	54	159	10.54
PrCG.R	Cingulum_Mid_R	12	−15	45	603	21.46
OFC	Frontal_Mid_R	39	21	36	724	10.51
	Occipital_Inf_L	−42	−81	−3	403	10.43

L, left; R, right; MNI, Montreal Neurological Institute.

### 3.4. Correlation results

**Figure 5:**
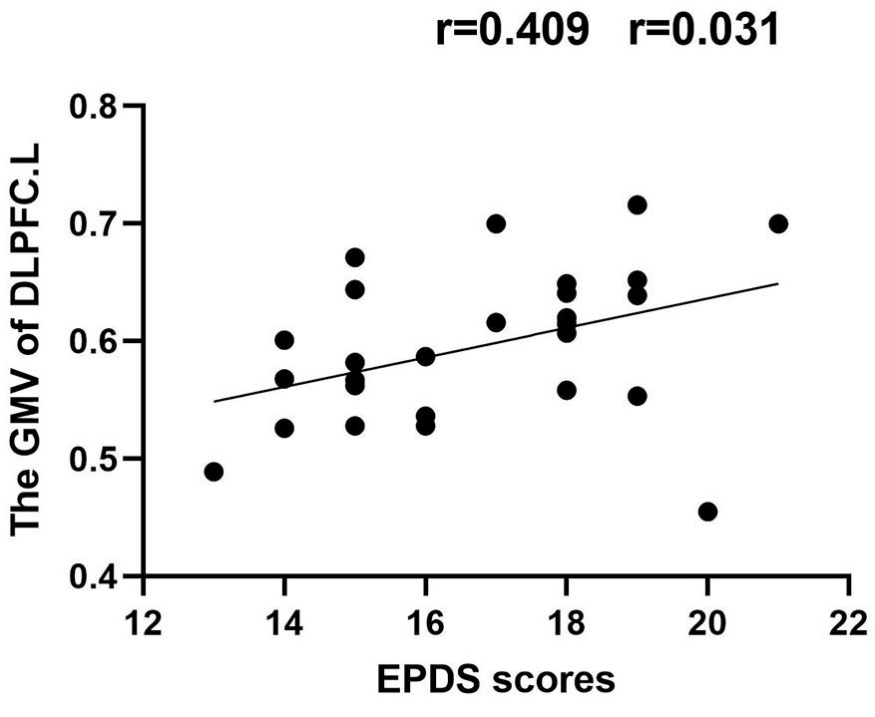
Correlation between GMV_DLPFC.L_ and EPDS scores (*r* = 0.409, *p* = 0.031).

**Figure 6:**
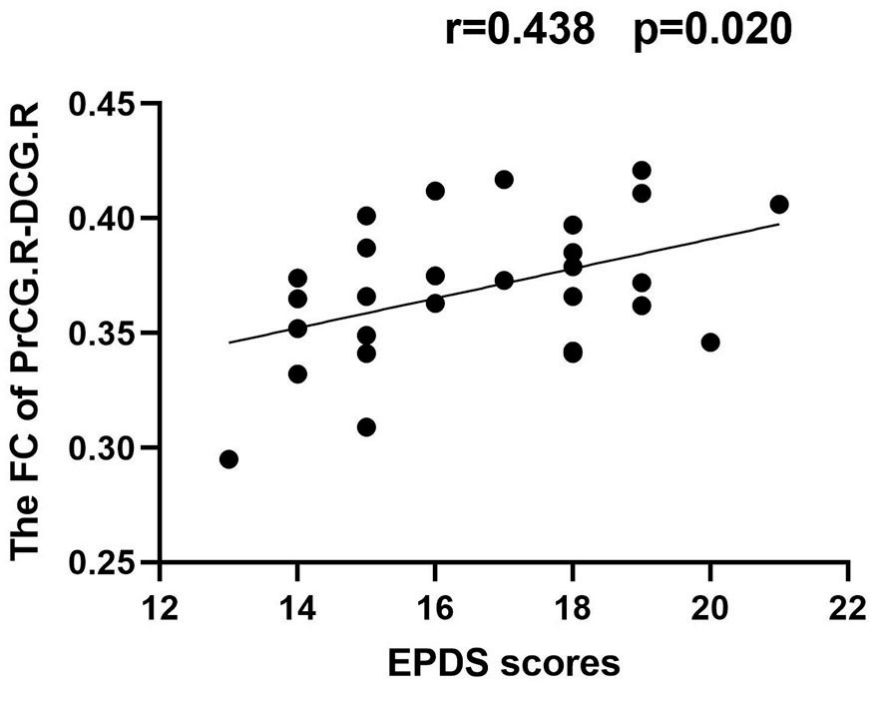
Correlation between FC_PrCG.R−DCG.R_ and EPDS scores (*r* = 0.438, *p* = 0.020).

In the PPD group, the GMV of DLPFC.L was positively correlated with EDPS scores (*r* = 0.409, *p* = 0.031); the FC of PrCG.R-DCG.R was positively correlated with EDPS scores (*r* = 0.483, *p* = 0.020) (Figures 5, 6).

The correlation analysis showed no correlation between the GMV of PrCG, R, and OFC with EDPS scores (GMV_PrCG, R_:*r* = −0.048, *p* = 0.807; GMV_OFC_: *r* = 0.204, *p* = 0.298 ). The FC of DLPFC.L-ACG.R, DLPFC.L-MFG.R, OFC-MFG.R, and OFC-IOG.L were not correlated with EDPS scores (FC_DLPFC.L−ACG.R_: *r* = 0.289, *p* = 0.135; FC_DLPFC.L−MFG.R_:*r* = 0.357, *p* = 0.062; FC_OFC−MFG.R_: *r* = 0.322, *p* = 0.094; FC_OFC−IOG.L_: *r* = 0.259, *p* = 0.183).

The correlation analyses indicated no correlation between the GMV of DLPFC.L, PrCG,R, and OFC with PSQI scores (GMV_DLPFC.L_: *r* = 0.141, *p* = 0.473; GMV_PrCG,R_: *r* = 0.041, *p* = 0.835; GMV_OFC_: *r* = 0.077, *p* = 0.698 ). The FC of DLPFC.L-ACG.R, DLPFC.L-MFG.R, PrCG.R-DCG.R, OFC-MFG.R, and OFC-IOG.L were not correlated with PSQI scores. (FC_DLPFC.L−ACG.R_: *r* = 0.209, *p* = 0.286; FC_DLPFC.L−MFG.R_: *r* = 0.217, *p* = 0.266; FC_PrCG.R−DCG.R_:*r* = 0.276, *p* = 0.155; FC_OFC−MFG.R_:*r* = 0.186, *p* = 0.344; FC_OFC−IOG.L_:*r* = 0.358, *p* = 0.061).

## 4. Discussion

The present study applied combined VBM and seed-based FC methods to observe whole-brain FC changes and discover the link between structural and functional abnormalities in PPD patients, and the three notable findings include those as follows: (1) The VBM analysis indicated increased volume in the regions of the DLPFC.L, the PrCG.R, and OFC in patients with PPD compared to HPW. (2) Compared with HPW, the PPD group showed significantly increased FC between the DLPFC.L and ACG.R, the DLPFC.L and MFG.R, the right PrCG and DCG.R, the OFC and MFG.R, and the OFC and IOG.L in the seed-based FC analysis. (3) The GMV of DLPFC.L and the FC between PrCG.R and DCG.R had a positive correlation with EPDS scores.

The DLPFC is a core brain region of the executive control network (ECN), which plays an essential role in complex cognitive functions and participates in the cognitive regulation of behavior and emotion (Causse et al., 2022). The prefrontal cortex (PFC) has been associated with stress, mood disorders, and parenting behaviors (Grande et al., 2021). A study showed that changes in the GMV of DLPFC were associated with stress and anxiety (Bao et al., 2022). The DLPFC.L is an essential clinical treatment target of some cognitive impairment and emotional disorders, and patients were found that their symptoms such as anxiety and depression of patients were improved after repetitive transcranial magnetic stimulation (rTMS) in the DLPFC.L (Cox et al., 2020; Klooster et al., 2020). In this study, we found that the GMV of the DLPFC.L was increased in PPD patients, which was consistent with previous research (Cheng B. et al., 2022). It may indicate that increased GMV of the DLPFC.L is a potential neurobiological manifestation of PPD. We speculated that when PPD patients could not withstand long-term parenting stress and anxiety, the GMV of DLPFC.L might increase and further cause worse parenting behaviors. Otherwise, we also found a positive correlation between increased the GMV of DLPFC.L and EPDS scores, which may support our previous speculation. The seed-based FC analysis showed that DLPFC.L presented more robust connectivity with the ACG.R and MFG.R than with HPW. The ACG plays an essential role in the cognitive functions of conflict monitoring and attentional control, particularly in regulating perception and working memory processes (Meroiti, 2022). The MFG is a crucial area facilitating attentional processes and plays an important role in the reorienting of attention, working memory, and speech and language comprehension (Briggs et al., 2021). The DLPFC is mainly involved in cognitive, sensory processing, and emotional regulation. The FC between the DLPFC.L and ACG.R and also between DLPFC.L and MFG.R increased in PPD patients, which may be related to some clinical behavioral symptoms such as mood disorders and attention and working memory deficits. PPD patients may pay less attention to normal emotional and physical demands of their infants. For PPD patients, relevant structural and functional changes of DLPFC may be an important reason for maladaptation and interactive behavior barriers with infants during the postpartum period.

The PrCG is a core component of the sensorimotor network (SMN), which is a brain region responsible for controlling voluntary movements and associated with behavioral performance, goal control, and motor function (Zhu et al., 2022). A study found that abnormal changes in the PrCG were associated with psychomotor disorders and poor action planning (Walther and Mittal, 2022). Moreover, another study found that aberrant activation of the PrCG was associated with suicide risk in patients with mood disorders (Harms et al., 2019). In the present study, we found that PPD patients had increased GMV in the PrCG.R and increased FC between PrCG.R and DCG.R compared with HPW, and the FC between PrCG.R and DCG.R was a positive correlation with EPDS scores. The DCG.R belongs to the cingulate gyrus, which associates with emotion regulation, cognitive processing, motor behavior, and internal sensory adaptation (Zauli et al., 2022). One study found that abnormal FC in DCG in PPD patients may be associated with depressive symptoms (Zhang X. et al., 2022). In PPD patients, the strength of FC between PrCG.R and DCG.R was possibly related to the severity of depressive symptoms. We speculated that, with the aggravation of depressive symptoms, negative thinking and rumination might be repeated in the brain of PPD patients, eventually leading to emotional breakdowns and uncontrolled behavior. Abnormalities of PrCG.R would cause SMN network disorders and then lead to PPD patients being unable to control their behaviors under highly negative emotions, resulting in severe aggressive behaviors such as infanticide, self-mutilation, and suicide.

The OFC is a critical brain region for emotion regulation and plays a significant role in maintaining reward, emotional states online, and decision-making (Du et al., 2020). Multiple studies found that OFC is an indispensable brain region associated with emotions, especially depression (Rolls et al., 2020; Zhang S. et al., 2022). A previous study found that disrupted functional interhemispheric connectivity between bilateral OFC may be related to the vulnerability of decision-making in PPD patients (Zhang et al., 2020). In this study, the GMV of OFC was increased in PPD patients compared with HPW. The increased GMV of OFC may cause PPD patients hardly maintain long-term stable emotions and the reward feedback process of mother–child communication and correct decision-making. Meanwhile, FC results showed increased FC between the OFC and MFG.R and also OFC and IOG.L compared with HPW. The MFG.R is involved in some cognitive activities such as working memory and attention reorientation. The IOG is part of the occipital lobe, which is thought to play a role in visual recognition and episodic memory consolidation (Zhang et al., 2021). In PPD patients, the results of these FCs may be associated with impairments of emotional perception, attention, and memory.

The limitations of the present study are as follows: Firstly, we investigated the increased GMV in three brain regions and their enhanced FC with multiple brain regions in PPD. However, these results should be interpreted cautiously as this study did not involve a longitudinal study comparing gray matter differences in brain regions before and after PPD pregnancy. Secondly, the sample size of the present study is small. We will continue to collect cases in future to expand the sample size to validate the current findings. Finally, in addition to PPD gray matter volume, the links between other gray matter morphology (cortical thickness, surface area, mean curvature, etc.), white matter, and brain function can be further explored. They may reveal more detailed information about PPD neuropathology by observing the relationship between structural and functional levels in future.

## 5. Conclusion

This study provided information on brain structural and functional abnormalities in PPD patients by combining VBM and FC, which are potentially related to clinical manifestations. Otherwise, the increased GMV of DLPFC.L may be a unique structural pathological mechanism of PPD, which may be related to the inability of PPD patients to withstand long-term parenting stress. These findings enhanced the understanding of the neurobiological mechanisms of PPD and contributed to its more effective diagnosis and treatment.

## Data availability statement

The original contributions presented in the study are included in the article/supplementary material, further inquiries can be directed to the corresponding author.

## Ethics statement

All participants were informed about the procedures and details of the study and provided written informed consent. The study was approved by the ethical committee of the 960th Hospital of PLA Joint Logistic Support Force and Shandong Second Provincial General Hospital.

## Author contributions

CC contributed to the experiments, data analysis, and manuscript writing as the first author. BL and SZ contributed to performing the experiments and writing and revising the manuscript. ZL, YW, MX, YJ, SW, and GS contributed to the collection of patients. KL is the guarantor of this study, had complete access to all data in the study, and contributed to this study as the corresponding author. They accept responsibility for the integrity of the data and the accuracy of the data analysis. All authors contributed to the article and approved the submitted version.
